# A bibliometric analysis of research productivity in Parasitology by different world regions during a 9-year period (1995–2003)

**DOI:** 10.1186/1471-2334-6-56

**Published:** 2006-03-17

**Authors:** Matthew E Falagas, Paraskevi A Papastamataki, Ioannis A Bliziotis

**Affiliations:** 1 Alfa Institute of Biomedical Sciences (AIBS), Athens, Greece; 2 Alfa HealthCare, Athens, Greece; 3 Department of Medicine, Tufts University School of Medicine, Boston, Massachusetts, USA

## Abstract

**Background:**

The objective of this study was to estimate the research productivity of different world regions in the field of Parasitology.

**Methods:**

Using the PubMed database we retrieved articles from journals included in the "Parasitology" category of the "Journal Citation Reports" database of the Institute for Scientific Information for the period 1995–2003. Research productivity was evaluated based on a methodology we developed and used in other bibliometric studies by analysing: (1) the total number of publications, (2) the mean impact factor of all papers, and (3) the product of the above two parameters, (4) the research productivity in relation to gross domestic product of each region, and (5) the research productivity in relation to gross national income per capita and population of each region.

**Results:**

Data on the country of origin of the research was available for 18,110 out of 18,377 articles (98.6% of all articles from the included journals). Western Europe exceeds all world regions in research production for the period studied (34.8% of total articles), with USA ranking second (19.9%), and Latin America & the Caribbean ranking third (17.2%). The mean impact factor in articles published in Parasitology journals was highest for the USA (1.88). Oceania ranked first in research productivity when adjustments for both the gross national income per capita (GNIPC) and population were made. Eastern Europe almost tripled the production of articles from only 1.9% of total production in 1995 to 4.3% in 2003. Similarly, Latin America and the Caribbean and Asia doubled their production. However, the absolute and relative production by some developing areas, including Africa, is still very low, despite the fact that parasitic diseases are major public health problems in these areas.

**Conclusion:**

Our data suggest that more help should be provided by the developed nations to developing areas for improvement of the infrastructure of research.

## Background

Parasitology studies a broad group of infectious diseases with different incidence worldwide. Most of these diseases occur more commonly in areas with poor hygiene, where they represent a major public health problem [1,2]. In addition, many forms of parasites, especially those transmitted by vectors, can also infect humans regardless of the hygiene level of an area, thus representing a potential health problem also for people with good socio-economic status.

The worldwide community invests a considerable amount of resources for research in the field of Parasitology in order to cope better with parasitic diseases [3]. However, a bigger proportion of these resources should be probably spent in areas with high incidence of parasitic diseases and especially in regions where these diseases cause high morbidity and mortality, namely the developing countries. Although much progress is still needed, research in the field of Parasitology has led to many important advances in the management of these infections [4].

Thus, estimates of global and regional productivity of ongoing research in the field of Parasitology may be of interest. The purpose of our study was to evaluate the contribution of different world regions in scientific research in this field, as represented by the quantity and quality of published papers.

## Methods

### Journal selection

Journals were selected if they were included in both the "Parasitology" category of the Journal Citation Reports (JCR) database of the Institute for Scientific Information (ISI) [5] and in the electronic PubMed database [6]. The latest edition of JCR at the time of our analysis provided data until the year 2003. Moreover, the full address of the authors for several articles published prior to 1995 was not provided by PubMed. Thus, we examined the period 1995–2003.

### World division

Based on geographic, scientific and economic criteria [7] we divided the world into the following nine regions: Western Europe, Eastern Europe, United States of America (USA), Canada, Latin America and the Caribbean, Africa, Japan, Asia (excluding Japan), and Oceania. In our study Eastern Europe includes all formerly socialist economies of Europe plus Turkey. The rest of Europe plus Greenland is designated as Western Europe. Japan is studied separately from the rest of Asian countries. USA is studied together with Puerto Rico.

### Search strategy

In the search field of the PubMed database, we used a phrase consisting of four parts joined together by the so-called Boolean operators, i.e. AND, OR, and NOT. In addition, we limited each search to a specific year by using the "Limits" function, which is incorporated in the search engine. Finally, we used the characterization "journal article [pt]" in the search field of the database ("pt" designates publication type), in order to include only original articles and reviews, excluding publication types, such as letters, editorials, and news reports.

For example, in order to search for articles published in the "International journal for parasitology" and whose first author's address was in Western Europe, we used the following text: *International journal for parasitology [journal] AND journal article [pt] AND (Belgium [AD] OR Denmark [AD] OR Danish [AD] OR Copenhagen [AD] OR*...*) NOT (Australia [AD] OR South Africa [AD] OR USA [AD] OR Ukraine [AD] OR*...*)*. In the first parenthesis of the search phrase, the countries of the implicated region are included. In some articles' addresses, only cities or areas were registered but not the name of the country, thus, to widen our search criteria, some big and/or capital cities (e.g. *Munchen, London*, or *Moscow*) and all the individual states of USA were also included in the first parenthesis of the search phrase accordingly. In the second parenthesis, after the word NOT, certain addresses are excluded in order to avoid double counting. For example, "New England, USA" (a part of the USA) may be counted in searches of articles originating from both USA and Western Europe, where England is located. To avoid such mistakes, we checked many of our searches and added exclusion criteria. These were included in the second parenthesis of our search string, i.e. when searching for Western Europe, we added: NOT *(Australia [AD] OR South Africa [AD] OR USA [AD] OR*...*)*.

Subsequently, the results of these searches (the number of articles produced by each world region in a specific journal within a year) were summed up. For confirmation purposes, the sum of articles produced by all different world regions in a journal was compared to the actual total number of articles published in that journal for a specific year. This number was obtained from PubMed without using any address limits. This way we identified unretrieved addresses and thus improved our search methodology. Despite our efforts, some articles were missed because the full address was not registered. We assumed that the numerical error was not significant, if less than 5% of the total articles of a specific journal during a year had missing addresses. If more than 5% of the total articles of a specific journal during a year, had missing addresses, we performed searches for the author's address by checking other articles of the same author within the same or closest possible year.

To strengthen the methodological validity of our study, two independent investigators conducted the data collection. In cases of disagreement between the two investigators the results were discussed in meetings of all authors.

### Indices of research productivity

The number of published articles was considered as an index of quantity of research productivity. The mean impact factor of the published articles was considered as an index of quality of research productivity. Finally, the product of the number of articles published in a journal multiplied by the impact factor of the journal, for the year studied, was used to evaluate the combined quantity and quality of research productivity. The sum of these products from all journals for each world region within a year was designated as the "total product" for each region within the studied year.

To further evaluate factors associated with the research published in Parasitology journals, we used relevant "World Development Indicators" [8] from the online databases of the World Bank. The research productivity of different world regions was evaluated in relation to total population, gross domestic product (GDP) in standard 1995 US dollars, and gross national income (GNI) per capita (Atlas method).

## Results

Eighteen journals met the inclusion criteria and were consequently included in our study (Table 1). During the study period 18,377 articles were published in these journals, of which 18,110 (98.6% of all) were identified by our searches and categorized to their respective regions of origin. The absolute and relative production of articles by each world region, as well as the respective mean impact factor of the articles per region are presented in the Additional file 1 (see Additional file 1). Western Europe was by far the most productive area in the field of Parasitology, with 34.8% of all articles (6,302 articles) coming from this area. USA ranked second (3,599 articles, 19.9% of total) and Latin America and the Caribbean third (3,111 articles, 17.2% of total). The mean impact factor of all retrieved articles in the study period was 1.60, with articles coming from the USA having the highest (1.88) and articles from Oceania having the second highest (1.86) mean impact factor.

**Table 1:** Journals analysed in the study

**Journal title**	**Years included**	**Language**
*Acta tropica*	1995–2003	English
*Advances in parasitology*	1995–2003	English
*Annals of tropical medicine and parasitology*	1995–2003	English
*Experimental parasitology*	1995–2003	English
*Folia parasitologica*	1995–2003	English
*International journal for parasitology*	1995–2003	English
*Journal of helminthology*	1995–2003	English
*Journal of parasitology*	1995–2003	English
*Memorias do Instituto Oswaldo Cruz*	1995–2003	Portuguese, English, French, or Spanish; some summaries in these languages and German.
*Molecular and biochemical parasitology*	1995–2003	English
*Parasite : journal de la Société française de parasitologie*	1995–2003	Articles in English and French, with summaries in both languages
*Parasite immunology*	1995–2003	English
*Parasitology*	1995–2003	English
*Parasitology international*	2002–2003	English
*Parasitology research*	1995–2003	English
*Systematic parasitology*	1999–2003	English
*Trends in parasitology (Parasitology today)*	1995–2003	English
*Veterinary parasitology*	1995–2003	English

Some regions increased their absolute and relative production during the study period. Eastern Europe almost tripled the production of articles; from only 1.9% of total production in 1995 its production reached 4.3% of total in 2003. Similarly, Latin America and the Caribbean and Asia doubled their production and, although in 1995 they represented 12.7% and 5.7% of the total production respectively, in 2003 they reached 19.0% and 9.0% of total production. Western Europe increased the number of articles by about 150 annually but remained constantly around 35% of total production. In the contrary, the USA had almost the same number of articles annually throughout the study period, and consequently its relative contribution to the field of Parasitology fell from 23.6% of total production in 1995 to 17.5% in 2003. Moreover, since 2001 USA fell on the relevant list, ranking third in the number of articles produced, coming after Latin America and the Caribbean which ranked second.

**Figure 1:**
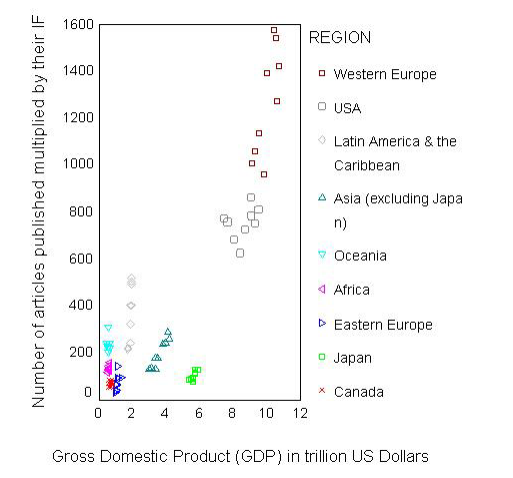
Scatter plot depicting the relationship of the annual "total product" of research productivity (number of articles published multiplied by their impact factor) in the field of Parasitology, for different world regions, for the period 1995–2003, with the gross domestic product (GDP) in trillion of 1995 US dollars.

Figure 1 presents estimates for quality and quantity of published research articles in relation to the gross domestic product (GDP). As shown, scientific production in the field of Parasitology is not strictly linked to the GDP of a region. For example Japan has higher GDP than the rest of Asia and Latin America and the Caribbean but the opposite is true for their respective research production in this field. In addition, although Western Europe during some years had similar GDP with the USA, it achieved a higher total research product during all study years.

Table 2 presents the quality and quantity of published research adjusted for the population and the gross national income per capita (GNIPC) of the region. Specifically, it shows the ratio of scientific "total product" per capita divided by the gross national income per capita for each region annually and for the whole study period. Oceania outweighs in production all other areas when adjustments for both GNIPC and population were made. Africa ranks second and Latin America and the Caribbean third. On the other hand, Japan exhibits the lowest productivity, having the highest GNIPC compared to all other areas.

**Table 2:** Research output of different world areas, published in journals included in the category of "Parasitology" of the Institute for Scientific Information (ISI), adjusted for population and gross national income per capita (GNIPC).

	**Number of publications multiplied by the impact factor per million population divided by the GNIPC (in 10,000 1995 US dollars)**									
	**1995**	**1996**	**1997**	**1998**	**1999**	**2000**	**2001**	**2002**	**2003**	**Average**
Oceania	5.0	5.1	4.6	4.0	5.8	5.8	4.2	3.8	4.2	4.7
Africa	2.6	2.5	2.8	2.6	2.6	2.3	2.1	2.7	2.2	2.5
Latin America & the Caribbean	1.3	1.3	1.3	1.7	2.2	2.1	2.5	2.6	2.7	2.0
Western Europe	1.1	1.1	1.2	1.0	1.4	1.5	1.5	1.2	1.3	1.3
Canada	1.0	1.3	1.4	1.2	0.9	0.9	1.2	1.0	1.0	1.1
USA	1.1	1.0	0.9	0.8	0.8	0.9	1.0	0.8	0.9	0.9
Eastern Europe	0.3	0.5	0.4	0.7	0.6	0.8	1.2	0.7	0.8	0.7
Asia (excluding Japan)	0.4	0.4	0.5	0.4	0.5	0.6	0.6	0.6	0.7	0.6
Japan	0.2	0.2	0.2	0.1	0.2	0.2	0.2	0.2	0.3	0.2

## Discussion

Our analysis provides some estimates of research productivity of different world regions in the field of Parasitology. Western Europe leads the world regarding the scientific production of research papers in this important biomedical field for global health. This may be partially explained by the fact that Western Europe has a long tradition in the study of diseases of interest for the tropics. In addition, our data show that the relative contribution of the USA in Parasitology, as proportion of the global production, fell gradually during the nine-year study period. Our results are in keeping with results from other studies of bibliometric research in fields other than Parasitology [9,10]. It should be pointed out that the data do not show an absolute decrease in the scientific production from the USA but rather a larger relative increase in research production by other regions.

It is reassuring that developing areas of the world such as Latin America and the Carribean and, to a lesser extent, Asia produce a considerable proportion of the worldwide research production in the field of Parasitology. This may be explained by the fact that economies of these regions are gradually improving; in addition, it may reflect increased research collaborations between countries in these areas and developed countries.

It is interesting that Oceania produced more scientific research in the field of Parasitology compared to other world regions, including the USA and Western Europe, when adjustments for the GNIPC and the population of the area were made. Oceania has also ranked high in other bibliometric studies of different biomedical fields that we performed using the same methodology [10,11], when both the aforementioned adjustments were made, or when only adjustment for population was made [12]. This fact may reflect the high priority that has been given to scientific research in Oceania. Furthermore, Africa and Latin America and the Carribean also ranked very high when adjustments for the GNIPC and the population of the area were made. The high ranking of Africa on this list, is mainly due to the very low GNIPC and not due to the large research productivity in Parasitology of this continent. However, it is reassuring to note that a considerable amount of publications coming from Africa is the result of multinational collaborations [13].

The study is not without limitations, most of which are the same with those of the studies we performed in other biomedical fields [10,11]. First of all, we used JCR criteria for including medical journals in the study. Articles published in non JCR-cited journals were not included, although they contribute to scientific production [14]. In addition, we searched journals included only in the "Parasitology" category of the JCR, although many articles regarding parasitic diseases are published in journals of other JCR categories, with wider field of interest, such as "Medicine, General and Internal", "Medicine, Research and Experimental", and "Infectious Diseases". Furthermore, when interpreting estimates regarding the quality of published, one should take into account that the JCR impact factor has often been criticized as a tool for measuring scientific research quality [15-17]. Yet, thus far it has not been replaced by any other worldwide-accepted method. JCR uses several criteria in order to include a journal in its databases, and for half a century the impact factor represents the best method of biomedical journal categorization [18,19].

Another limitation is that articles in PubMed have only the address of the first author registered; thus, it was not possible to estimate the quantity of articles that resulted from multinational/multiregional collaborations. This may cause some problems when estimating research productivity in developing regions like Africa, where multinational collaborations are not uncommon for conduction of research in parasitology. Furthermore, the search system we created was not able to retrieve the addresses of all articles indexed in PubMed. However, we managed to retrieve 98.5% of all published articles in the field, therefore we assumed that the number of missed articles did not significantly affect our study results. Finally, we should emphasize that the division of the world into different regions could be done in various ways. Our classification was based on several criteria, but alternative approaches would also be appropriate. For example, Canada could be grouped together with USA, and Japan could be studied together with the other Asian countries. We believe that the categorization we used takes into account geographic, economic, and, most importantly, scientific criteria (i.e., Canada and Japan represent powerful autonomous scientific world regions).

## Conclusion

In conclusion, we estimated the research productivity in the field of Parasitology by different world regions during a nine-year period. Our study provides data that may be used by funding agencies and governmental bodies regarding the development of networks of research between developing and developed countries. It seems that such help related to the infrastructure of biomedical research will benefit more those that mainly need it, i.e. the citizens of developing countries.

## Competing interests

The author(s) declare that they have no competing interests.

## Authors' contributions

MEF conceived the idea for the study; IAB and PAP collected the data; MEF drafted the manuscript. IAB and MEF contributed in the writing and preparation of the manuscript. All authors read and approved the final manuscript.

## Pre-publication history

The pre-publication history for this paper can be accessed here:



## Supplementary Material

Additional File 1 Number of articles published in journals included in the field of Parasitology category of "Journal Citation Report" database and indexed by PubMed, from different world regions, for the period 1995–2003. The absolute and relative production of articles by each world region, as well as the respective mean impact factor of the articles per region, is presented in the Additional file 1. The mean impact factor of all retrieved articles in the study period was 1.60, with articles coming from the USA having the highest (1.88) and articles from Oceania having the second highest (1.86) mean impact factor. Click here for file
