# Supplementary material for: A bibliometric analysis of research productivity in Parasitology by different world regions during a 9-year period (1995–2003)
Source: BMC Infect Dis. 2006 Mar 17;6:56. doi: 10.1186/1471-2334-6-56 (PMC1431540; doi:10.1186/1471-2334-6-56)
Supplement: Additional File 1 — Number of articles published in journals included in the field of Parasitology category of "Journal Citation Report" database and indexed by PubMed, from different world regions, for the period 1995–2003. The absolute and relative production of articles by each world region, as well as the respective mean impact factor of the articles per region, is presented in the Additional file 1. The mean impact factor of all retrieved articles in the study period was 1.60, with articles coming from the USA having the highest (1.88) and articles from Oceania having the second highest (1.86) mean impact factor. [file 1471-2334-6-56-S1.pdf]

Additional File 1. Number of articles published in journals included in the field of Parasitology category of "Journal Citation Report" database and indexed by PubMed, from different world regions, for the period 1995-2003.

	1995		1996		1997		1998		1999		2000		2001		2002		2003		*1995-2003*		
**WORLD AREAS**	Number of articles (% percentage within a calendar year)																				***Mean impact factor (1995 - 2003)***
**Western Europe**	599	(35.7)	626	(35.3)	689	(36.2)	660	(34.2)	704	(35.1)	806	(36.7)	776	(35.0)	681	(31.4)	761	(33.9)	*6302*	*(34.8)*	*1.80*
**USA**	397	(23.6)	418	(23.6)	393	(20.7)	372	(19.3)	388	(19.4)	417	(19.0)	451	(20.4)	371	(17.1)	392	(17.5)	*3599*	*(19.9)*	*1.88*
**Latin America & the Caribbean**	213	(12.7)	233	(13.2)	258	(13.6)	353	(18.3)	384	(19.2)	361	(16.4)	407	(18.4)	475	(21.9)	427	(19.0)	*3111*	*(17.2)*	*1.07*
**Asia (excluding Japan)**	96	(5.7)	104	(5.9)	137	(7.2)	112	(5.8)	135	(6.7)	170	(7.7)	161	(7.3)	198	(9.1)	202	(9.0)	*1315*	*(7.3)*	*1.37*
**Oceania**	145	(8.6)	142	(8.0)	143	(7.5)	137	(7.1)	157	(7.8)	137	(6.2)	104	(4.7)	106	(4.9)	108	(4.8)	*1179*	*(6.5)*	*1.86*
**Africa**	103	(6.1)	99	(5.6)	119	(6.3)	125	(6.5)	95	(4.7)	111	(5.1)	84	(3.8)	121	(5.6)	107	(4.8)	*964*	*(5.3)*	*1.27*
**Japan**	57	(3.4)	62	(3.5)	63	(3.3)	57	(3.0)	51	(2.5)	72	(3.3)	81	(3.7)	86	(4.0)	113	(5.0)	*642*	*(3.6)*	*1.49*
**Eastern Europe**	32	(1.9)	39	(2.2)	43	(2.3)	63	(3.3)	56	(2.8)	83	(3.8)	109	(4.9)	91	(4.2)	96	(4.3)	*612*	*(3.4)*	*1.11*
**Canada**	37	(2.2)	48	(2.7)	58	(3.0)	50	(2.6)	33	(1.6)	39	(1.8)	42	(1.9)	42	(1.9)	37	(1.7)	*386*	*(2.1)*	*1.69*
Total	*1679*		*1771*		*1903*		*1929*		*2003*		*2196*		*2215*		*2171*		*2243*		*18110*		
